# Narrative Review of Open Abdomen Management and Comparison of Different Temporary Abdominal Closure Techniques

**DOI:** 10.3389/jaws.2025.14119

**Published:** 2025-06-10

**Authors:** Levent Afsar, Paolo Carlo Capelli, Gessica Carvalho, Deepanjan Ghosh, Wendy Ofosu, Christian Seelandt, Sadhana Trivedi

**Affiliations:** ^1^ Solventum Turkey, Istanbul, Türkiye; ^2^ Solventum Italy, Milan, Italy; ^3^ Solventum Brazil, Campinas, Brazil; ^4^ Solventum Corporation, Maplewood, MN, United States; ^5^ Solventum United Kingdom, Leicestershire, United Kingdom; ^6^ Solventum Germany, Wiesbaden, Germany; ^7^ Solventum Ireland, Dublin, Ireland

**Keywords:** open abdomen, temporary abdominal closure, fascial closure, enteroatmospheric fistula, negative pressure therapy

## Abstract

**Introduction:**

The management of an abdomen kept open after life-saving intervention for patients with intra-abdominal hypertension or abdominal compartment syndrome (ACS) constitutes an inevitable measure. Various temporary abdominal closure (TAC) techniques have been developed to reduce complications, facilitate re-entry for subsequent procedures, and support improved clinical outcomes. The goal is timely, definitive closure, promoting long-term patient health with full re-establishment of abdominal wall integrity.

**Methods:**

This review details TAC techniques and synthesizes guidelines from leading medical organizations. It examines key studies on open abdomen management, identifies research gaps, and proposes future research directions.

**Results:**

The TAC techniques include skin closure with clips or sutures, mesh closure and dynamic retention sutures, Wittmann Patch, Bogota Bag, Barker Vacuum Pack, and commercial negative pressure therapy (NPT) systems. Leading organizations such as the *World Society of the Abdominal Compartment Syndrome* and the *Eastern Association for the Surgery of Trauma* recommend NPT systems due to their superior clinical results. These systems reduce incidences of ACS, promote primary fascial closure, and decrease mortality.

**Recommendations:**

Successful management of the open abdomen requires tailored TAC technique selection to meet specific patient needs while considering available resources. Though commercial NPT systems provide better long-term outcomes, traditional methods like the Barker Vacuum Pack remain viable in resource-constrained environments. Future research should prioritize cost-benefit analyses to ensure that high-quality care is aligned with superior clinical outcomes.

**Conclusion:**

The document highlights the importance of early diagnosis and closure and emphasizes the need for further studies to optimize surgical techniques and improve cost-effectiveness.

## Introduction

The open abdomen (OA), also known as laparostomy, is an intervention that leaves all layers of the abdominal wall open for several critical reasons including severe peritonitis, sepsis, abdominal trauma, damage control surgery, vascular emergencies, abdominal wall necrosis, abdominal compartment syndrome (ACS), and acute mesenteric ischemia [1–3]. The benefits from this intervention include facilitating a second look and avoiding or managing ACS. The OA can be a lifesaving procedure in patients with intraabdominal hypertension (IAH) or ACS [2, 4]. Temporary abdominal closure (TAC) techniques are utilized to contain the abdominal content, prevent abdominal cavity contamination, protect the viscera from desiccation and evisceration, facilitate peritoneal fluid evacuation, allow easy access to the abdominal cavity, and reduce edema [5]. The ideal temporary abdominal closure technique (TAC) should be easy to use, require limited dressing changes, prevent abdominal fascia retraction, avoid recurrent ACS, reduce the risk of complications and mortality, and still be relatively cost-effective [6, 7]. This narrative review explores the current understanding of OA management, compares TAC techniques, and discusses relevant guidelines and consensus recommendations.

## Open Abdomen Management: Concepts and Challenges

Laparostomy can be purposely created by leaving an abdominal incision open following surgery or by opening or reopening the abdomen due to ACS. The recommendation is to achieve primary fascial closure as soon as possible [8]. Based on this premise, early definitive closure of the abdominal wall should be the main objective of any strategy for OA management once the causes that originated it have been resolved. The consequences of not closing an abdomen in a timely manner can lead to frozen or hostile abdomens as well as other complications such as protein and fluid loss, intestinal fistulation, and loss of abdominal domain. Hence, the main goals of TAC techniques are to limit loss of domain, facilitate fascial closure, manage fluid loss, and avoid further complications [3, 9, 10]. Strategies to achieve these goals have evolved over time, with the development of various TAC techniques.

## Review of Temporary Abdominal Closure Techniques

Different techniques have been used for TAC in the past decades, including skin approximation with towel clips, meshes, the Wittman patch, Bogota bag, Barker vacuum pack, and commercial negative pressure therapy. Each technique has its own advantages and disadvantages (Table 1) [10, 11].

**TABLE 1:** Comparison of temporary abdominal closure techniques and devices.

Technique	Advantages	Disadvantages	Evidence summary
Bogota Bag	• Readily available, can be used in emergency settings when no other option available • Prevents evisceration of abdominal contents	• Higher risk of infection, due to inability to manage fluids • Does not prevent temperature shifts • Does not prevent abdominal domain loss • Does not allow monitoring of fluid output or intraabdominal pressure	Clinical evidence shows inferior results for intra-abdominal pressure management, compared to NPT [28, 29]
Velcro or zipper-type synthetic materials, e.g., Wittmann patch	• Provides medial tension • Allows progressive tension closure • Preserves abdominal wall domain • Prevents evisceration of abdominal contents	• Complicated application technique • Does not provide easy removal of intraperitoneal fluid • Does not prevent adhesion between peritoneum and bowel	High primary fascia closure rates (75%–90%) [2]
Synthetic Meshes	• Provides barrier • Prevents evisceration of abdominal contents • Preserves abdominal domain • Provides medial tension • Helps gradual safe definitive fascial closure	• Risk of hernia, infection • Risk of abdominal wall/viscera adherences	Effective for protection and prevention of fascial retraction, but wrinkling, infection, hernia and enteric fistulas may arise [30]
Dynamic retention suture, ABRA	• Prevents retraction of fascia • Helps gradual safe definitive fascial closure • Avoids the need for multiple returns to OR	• Complicated application technique • High risk of complications • Risk of hernia development	Consensus shows satisfactory rates of fascial closure/delayed closure, but risk of hernia development is a concern [31]
Barker’s Vacuum Packing Technique	• Prevents evisceration of abdominal contents • Helps in removal of intraperitoneal fluid • Preserves abdominal domain • Helps definitive fascial closure • Provides medial tension	• Does not prevent adhesion between peritoneum and bowel • High risk of fistula • Requires frequent changes • Poor outcomes • Labor-intensive • Does not allow monitoring of fluid output and pressure	Option in resource-limited settings, higher complication rates, higher mortality and reduced fascial closure compared to commercial NPT systems [20]

Commercial Negative Pressure Therapy Devices			
Device	Features	Considerations	Summary of Clinical Evidence
3M™ AbThera™ Therapy (Solventum Corporation, Maplewood, MN)	• Standardized NPT • Provides barrier • Prevents evisceration of abdominal contents • Effectively removed intraperitoneal fluid • Preserves abdominal domain • Provides medial tension • Helps provide gradual, safe definitive fascial closure	• Not widely available in limited resource settings	Superior outcomes in terms of primary closure, shorter OA duration, reduced mortality, better fluid management [21, 28, 32]
Renasys AB Abdominal Dressing Kit	• Provides barrier • Prevents evisceration of abdominal content • Provides fluid evacuation • Similar with other commercial NPT systems	• Design not suitable to remove fluid from deep within paracolic gutters	Lacks high quality clinical evidence
Invia Abdominal Dressing Kit	• Similar with other commercial NPT systems • Ease of application • Portable • User-friendly interface	• Requires specialized devices	Lack of published specific OA closure rates
Suprasorb® CNP Drainage Film	• Similar with other commercial NPT systems		Lack of published specific OA closure rates
XLR8 Abdominal Dressing Kit	• Provides fluid evacuation • Similar to other commercial NPT systems		Lack of clinical data and published OA closure rates
Avance Abdominal Dressing Kit	• Similar with other commercial NPT systems	• Design not suitable to remove fluid from deep within paracolic gutters	Lack of clinical data and published OA closure rates
Confort Open Abdominal Closure Set	• Similar with other commercial NPT systems	• Design not suitable to remove fluid from deep within paracolic gutters	Lacking high quality clinical evidence

NPT, Negative pressure therapy; OA, Open abdomen; OR, Operating Room.

## Skin Only Closure With Towel Clips or Sutures

The temporary skin-only closure techniques use the skin to provide some abdominal wall stability with containment of abdominal viscera. These techniques use a series of towel clips or a rapid monofilament running suture. The towel clip closure is perhaps the most rapid of the temporary closure techniques. The closure types are swift, inexpensive, and easily available. The abdominal contents are maintained below the level of the fascia, which minimizes heat and fluid loss. On the other hand, there is a high risk of evisceration, injury and loss of skin, infection, and recurrent ACS since this technique does not allow fluid management and reduction in edema. High mortality associated with elevated intraabdominal pressure and subsequent ACS significantly increases the risk of mortality. As complications and higher risk of mortality has been associated with skin only closure, these techniques have been largely abandoned [12].

## Mesh Closure and Dynamic Retention Suture

Mesh closure techniques, including the use of absorbable and non-absorbable meshes, provide a physical barrier while allowing for gradual fascial closure. Dynamic retention sutures involve the placement of heavy sutures across the abdominal wall to prevent retraction of the fascia. Both methods are effective in achieving delayed primary closure but are associated with a higher risk of long-term complications such as hernia formation [12–14].

## Wittmann Patch

The Wittmann Patch, also called “artificial burr”, is a dynamic TAC system that involves a Velcro-like patch allowing gradual closure of the abdomen. The Wittmann Patch consists of two adherent Velcro sheets, one consisting of loops and the other of hooks. An adhesion prevention barrier is inserted between the bowels and peritoneum deep into lateral gutters. The sheets are cut to the length of the incision and sewn to the fascia. The sheets are then pulled from either side, allowing them to overlap and be pressed together. Typically, the patch is sequentially tightened every 24–48 h until the fascia is approximately 2–4 cm apart. This temporary closure is removed at the final operation and some form of definitive closure is used to close the fascia primarily. This technique is particularly useful in patients requiring multiple re-operations, as it facilitates progressive tension closure. While it is more expensive than some of other methods, it offers superior outcomes in terms of primary fascial closure rates and reduced complications. The mesh, zippers, or Wittmann Patch permits rapid and safe reentry into the abdomen on re-exploration, and if an additional laparotomy is necessary in the future, permits a rapid closure. Other advantages of the Wittmann Patch technique include a gradual approximation of fascia, ease of re-exploration, and prevention of loss of abdominal domain. The Wittmann Patch technique is more costly and requires suturing to the abdominal fascia, which may increase the risk of fascial trauma and necrosis, and future incisional hernias may develop. Finally, this technique does not effectively evacuate peritoneal fluid, this would be a major concern for non-trauma patients [12, 15, 16].

## BOGOTA BAG (ALSO KNOWN AS THE BORRÁEZ BAG)

The Bogota bag method consists of suturing a sterile irrigation bag to the fascia or to the skin and leaving fascial edges intact. This technique permits the release of intraabdominal pressure, preventing evisceration, and it is cheap and easy to apply. However, this “non-traction technique” permits fascial retraction with loss of domain, making definitive closure harder and increasing the risk of larger incisional hernia development. Furthermore, the Bogota Bag technique does not provide effective removal of intraperitoneal fluid, which is a limitation when dealing with intraabdominal sepsis. Other disadvantages are higher risk of entero-atmospheric fistula (EAF), lower abdominal closure rates, and higher patient mortality [3, 17, 18].

## Barker’s Vacuum Pack Technique

The Barker’s vacuum pack technique, introduced in the 1990s, involves placing a non-adherent polyethylene sheet over the viscera, followed by moist surgical towels and adhesive drapes to create a seal. A suction drain is then applied to maintain a negative pressure environment. This method is simple, inexpensive, and widely used, particularly in resource-limited settings. Barker’s technique, once very popular, has been replaced with commercial negative pressure therapy systems based on the comparative studies favoring commercial NPT in terms of fascial closure, EAF formation,1 q and mortality [19–21].

## Commercial Negative Pressure Therapy (NPT) Systems

Several commercial negative pressure therapy (NPT) systems have been developed as alternatives to the Barker’s technique. Among all commercially available NPT systems, OA-NPT (3M™ AbThera™ Therapy, Solventum Corporation, Maplewood, MN) has the greatest number of published clinical evidence.

An advanced commercial NPT designed for OA management, OA-NPT works by transferring continuous negative pressure from the therapy unit to the perforated foam and encapsulated foam within the visceral protective layer. The negative pressure manifolds throughout the open abdomen facilitate the removal of exudate and infectious material and help reduce edema. At the same time, the perforated foam and the encapsulated foam collapse medially, drawing fascial edges closer together, which helps minimize fascial retraction and loss of domain, one important expectation from a commercial NPT in the OA would be the non-impaired stability of anastomoses under negative pressure [11, 21–23].

## Negative Pressure Technique Associated With a Dynamic Component

In general, negative pressure associated with a dynamic component (mesh-mediated fascial traction or dynamic sutures) allows to reach the best results in terms of delayed fascial closure, but dynamic sutures result more often in fistula [7].

## Guidelines and Consensus Recommendations

OA management has been the focus of several guidelines and consensus recommendations from surgical and critical care societies. These guidelines emphasize the importance of early recognition of the need for TAC, appropriate technique selection, and timely definitive closure.

1. World Society of the Abdominal Compartment Syndrome (WSACS): The WSACS guidelines recommend the NPT use as the preferred method for TAC due to its ability to reduce abdominal compartment syndrome, promote fascial closure, and decrease morbidity and mortality. The WSACS also highlighted the importance of achieving definitive closure within 7–10 days to minimize complications) [24]. WSACS suggests bioprosthetic meshes should not be routinely used in the early closure of the OA compared to alternative strategies and recommends measuring intraabdominal pressure (IAP) using the trans-bladder technique when any known risk factor for IAH/ACS is present in a critically ill or injured patient. The WSACS also recommends decompressive laparotomy in cases of overt ACS compared to strategies that do not use decompressive laparotomy in critically ill adults with ACS.
2. Eastern Association for the Surgery of Trauma (EAST): EAST guidelines advocate for the use of commercial NPT systems like OA-NPT for their superior outcomes in achieving primary fascial closure and reducing the incidence of enterocutaneous fistulae. However, they also acknowledge the role of traditional methods like Barker’s vacuum pack in resource-limited settings [25].
3. World Society of Emergency Surgery (WSES): The WSES guidelines recommend NPT as the preferred method for TAC, particularly in cases where there is a high risk of ACS. The guidelines emphasize the importance of achieving definitive fascial closure within 7–10 days to minimize complications such as fistula formation and abdominal wall retraction. In resource-limited settings, traditional methods like Barker’s vacuum pack remain valuable, but the use of commercial NPT systems is preferred where available [8].
4. European Hernia Society (EHS): The EHS guidelines highlight the importance of individualized patient care, with the choice of TAC technique guided by the patient’s condition, the need for re-exploration, and the risk of infection. The use of mesh-based TAC is generally discouraged in contaminated fields due to the risk of infection and long-term complications such as hernia formation. The guideline recommends that dynamic closure techniques should be prioritized over the use of static closure techniques [26].
5. National Institute for Health and Care Excellence (NICE): NICE recommends NPT for the OA according to the evidence for safety and efficacy. The guideline describes the patient profile for NPT in three groups: patients who have had surgery that did not involve the gastrointestinal tract, and in whom delayed primary closure is planned within about 1 week (for example, after 'damage-control’ surgery); patients who have had gastrointestinal tract surgery for the management of abdominal sepsis associated with severe gastrointestinal disease (including anastomotic dehiscence, visceral perforation or inflammatory bowel disease) or severe pancreatitis; and patients who have had abdominal wound dehiscence [27].

These guidelines consistently underscore the importance of a multidisciplinary approach to the management of the open abdomen, involving surgeons, intensivists, dietician and wound care specialists. The decision-making process should be guided by the clinical scenario, available resources, and the goal of early definitive closure to prevent complications.

## Discussion

Current organizational structure and information systems of healthcare delivery make it challenging to measure cost-effectiveness. Achieving high value for patients must become the overarching goal of healthcare delivery, with value defined as the health outcomes achieved per dollar spent [33]. Improving one outcome may affect other patient and healthcare cost outcomes. For example, timely treatment can shorten recovery, leading to reduced total healthcare costs across the patient’s care cycle. Since value is defined as outcomes relative to costs, measuring the total costs over a patient’s entire care cycle and weighing them against outcomes could enable true structural cost reduction, through steps such as reallocation of spending, better use of capacity, and shortening of length of stay.

The choice of which TAC technique to use depends on several factors, including the patient’s condition, the need for re-exploration, the risk of infection, and the available resources. Recent studies suggest that while commercial NPT systems are more effective in reducing complications and achieving primary fascial closure, reducing mortality risk, their high cost remains a significant barrier to widespread use. In contrast, traditional methods like the Barker’s vacuum pack and Bogota bag are more feasible due to available material but carry a higher risk of adverse outcomes and unfavorable prognosis. The potential morbidity of ventral hernia repair in this complex patient population should not be underestimated [34]. As treatment value for patients is often revealed only over time [4], the only way to accurately measure value is to track patient outcomes and costs longitudinally.

A retrospective review of 37 open abdomen patients with OA-NPT use between 2010 and 2011 were compared to 37 patients receiving OA management using the Barker technique between 2009 and 2010 [23]. This retrospective review demonstrated a significant increase in the success of fascia closure using the OA-NPT technique, compared with the Barker technique. While OA-NPT did have a higher initial cost compared to Barker’s technique, the authors believe use of OA-NPT could provide cost savings due to a reduction of long-term morbidity and the cost of a subsequent ventral hernia repair.

## Conclusion

The management of the OA requires a tailored approach, with the choice of TAC technique guided by patient-specific factors and available resources. While commercial NPT systems offer the most promising outcomes, their high cost and resource requirements limit their use in many geographies. A comprehensive value-based approach showing their cost-effectiveness is needed. Traditional methods like the Barker’s vacuum pack remain as an option, particularly in resource-limited environments when other alternatives are not available, despite their higher complication rates and worse outcomes. Guidelines and consensus recommendations emphasize the importance of early recognition, appropriate technique selection, and timely definitive closure. Further research is needed to optimize these techniques and investigate cost-effectiveness that can be widely implemented across diverse healthcare settings.
